# Transfusion-transmitted parasitic infections

**DOI:** 10.4103/0973-6247.67018

**Published:** 2010-07

**Authors:** Gagandeep Singh, Rakesh Sehgal

**Affiliations:** *Department of Parasitology, PGIMER, Chandigarh-160012, India*

**Keywords:** Parasites, transfusion, transmitted

## Abstract

The transmission of parasitic organisms through transfusion is relatively rare. Of the major transfusion-transmitted diseases, malaria is a major cause of TTIP in tropical countries whereas babesiosis and Chagas’ disease pose the greatest threat to donors in the USA In both cases, this is due to the increased number of potentially infected donors. There are no reliable serologic tests available to screen donors for any of these organisms and the focus for prevention remains on adherence to donor screening guidelines that address travel history and previous infection with the etiologic agent. One goal is the development of tests that are able to screen for and identify donors potentially infectious for parasitic infections without causing the deferral of a large number of non-infectious donors or significantly increasing costs. Ideally, methods to inactivate the infectious organism will provide an element of added safety to the blood supply.

## Introduction

Although the incidence of blood transfusion-transmitted parasitic infections (TTPI) is lower in comparison to that of bacterial and viral infections, these organisms pose a considerable risk of illness, especially in immunocompromised individuals. As we know, bacterial contamination can occur at numerous points during the collection and the transfusion process but the TTPI are always donor derived. The most common parasitic organisms implicated in transfusion-transmitted infections are *Plasmodium* spp., *Trypanosoma cruzi*, *Babesia microti*, *Toxoplasma gondii*, *Leishmania* spp. etc. To be transmitted by blood transfusion, parasites must: (i) circulate in the blood stream of donors, (ii) comprise of certain physical characteristics and resist processing steps leading to the preparation of labile blood products (packed red blood cells, therapeutic frozen plasma, or platelet concentrates), (iii) survive conservation; further, to generate infection in the blood receiver, such parasites must retain infectivity.[1] This review discusses the etiological agents, epidemiology, and diagnostic modalities related to transfusion transmission of parasitic infections.

## Malaria

The first case of transfusion-transmitted malaria (TTM) was reported in 1911. Review of worldwide data recorded from 1911 to 1979 by Bruce-Chwatt[2] found that the reported incidence of TTM increased from 6 to 145 cases per year. In the early years, *P. vivax* was the most frequently reported species, but by the1950s *P. malariae* predominated, followed by *P. vivax*, *P. falciparum*, mixed infections, and *P. ovale*. In the 1970s, *P. vivax* was again the most common, followed by *P. malaria* and *P. falciparum*, with the proportion of the last increasing substantially.[3,4] In the USA before 1986, there were more cases of *P. vivax* than *P. falciparum*, but since then *P. falciparum* has been the single most common imported species. Indeed, the total for *P. falciparum* now exceeds that of the other three species combined. In 2004, 74% of imported malaria was caused by *P. falciparum*. The figures for imported malaria from 1985 to 1995 reported by other European centres also show a substantial proportion caused by *P. falciparum*– 82.2% in France and 59.4% in Italy compared with 38.5% in the USA over the same period.[5]

Transmission of malaria has been reported to occur mainly from single-donor products: red cells, platelets or white cell concentrates (because of contamination with residual red cells), cryoprecipitate, and from frozen red cells after thawing and washing. Transmission from single-donor fresh-frozen plasma has not been reported. Transmission from cryoprecipitate is rare and likely to reflect the preparation method and the degree to which the starting plasma is cell free.[6] There are two main aspects to bear in mind when considering malaria risk and transfusion: first, the malaria risk associated with any individual donor, and second, the ability of the systems to identify and manage the donor and any donation. It is here that there are fundamentally different approaches taken by different blood transfusion services: differences in the overall approaches taken between endemic and non-endemic countries and differences in the approaches taken between individual non-endemic countries.

Differentiation of cases of TTM from natural infections is very difficult in endemic areas as malaria occurring post-transfusion can be the result of natural infection via a mosquito bite, rather than from the transfusion received. Furthermore, in endemic areas, many of the donors and patients are already infected with, or are at high risk of, malaria infection. Identifying low-risk individuals is virtually impossible. One approach is to use Giemsa-stained thick films or rapid diagnostic tests (RDTs) for malarial antigen to identify those donors with higher levels of parasitaemia. However, it is clear that this approach only identifies the proportion of individuals with a parasitaemia above the detection limit for these techniques. It does not, however, prevent transmission from units of blood with parasitaemia too low to be detected by microscopy or RDTs. There are additional strategies that can be implemented. For example, the segregation of donations collected from low- and high-risk areas, with specific targeting of the donations from low-risk donor groups to low risk and the most vulnerable recipients. Routine antimalarial treatment of transfusion recipients is also performed in some areas. Such strategies are pragmatic approaches that are not absolute in their effectiveness, but can help to lessen the risk of TTM in such situations.[7]

However, in non-endemic countries the overall number of individuals with any malaria risk represents only a small proportion of the overall number of donors, the number of these donors is cumulative as, year-on-year, donors either visit malarious areas for the first time or individuals originally from malarious areas present as donors for the first time. Thus, there is a high reliance upon appropriate donor-deferral guidelines, and on the accuracy and clarity of the information gleaned from the donors about their travel and any consequent malaria risk.

Plasmodial infection that can be caused by “malaria” parasites after transfusion can thus be either free merozoites, intraerythrocytic forms of parasites or gametocytes, with the former two being infective but not the latter.[6] Of important note, those infective parasite stages are placed in contact with the host’s red blood cells while this host has not been “prepared” by a number of more or less efficient immune events. Very weak or no counteraction can then occur, causing rapid multiplication of infected red blood cells (iRBCs) responsible for morbidity and frequently mortality by complications such as organ failure rather than from acute anaemia.[8] Each of these steps even those which are quick can be counteracted by innate immune events which have started once the host is infected.

Apart from donor screening, other options to identify infected donors include use of tests primarily designed to detect parasites in symptomatic individuals or antibody screening tests. Tests for detection of parasites include thick/thin blood smears, fluorescent staining techniques, tests for circulating malarial antigen, or polymerase chain reaction (PCR) for detecting malarial DNA.[9–12] Examination of thick blood smears is not cost-effective for screening large numbers of donors, nor is it sensitive enough (limit of detection is approximately ten organisms/µL) to detect low levels of parasitemia that might exist in donors. Less than 50% of implicated donors in studies had positive smears, which is probably related to low levels of circulating parasites. Fluorescent stains such as acridine orange that stain nucleic acids can also be used for examination of a thick film for parasites or in systems that employ capillary tubes filled with blood e.g. quantitative buffy coat technique (QBC).[11]

Recently, there has been an increased use of immunochromatographic dipstick tests for rapid screening/diagnosis of acute malaria in endemic areas. These tests which use monoclonal antibody fixed to nitrocellulose strips detect circulating *P. falciparum* histidine-rich protein 2 (HRP-2) antigen or Plasmodium lactate dehydrogenase (pLDH). The level of detection required in acute cases is approximately 500 parasites/µL, which may be greater than the level of circulating parasites in an asymptomatic blood donor.[13]

On the other hand, serologic tests identify antibody-positive individuals, but do not indicate parasitemia because antibody levels can remain elevated up to 10 years after infection. In general, when used in a donor population with a low prevalence of malaria, antibody tests have poor positive predictive value. Laboratories in France, however, use an indirect fluorescent antibody (IFA) method to detect antibodies to Plasmodium sp. in at-risk donors. There has not been a reported case of transfusion-transmitted malaria since the test was instituted in 1994.[14] One study evaluating an enzyme-linked immunosorbent assay (ELISA) test that used *P. falciparum* antigen indicated that the test might not be sensitive and specific enough to screen for antibodies in at-risk donors whose medical history indicated they might have been exposed to malaria.

Silvie *et al*, used a combination of a *P. falciparum* HRP-2 antigen test and an enzyme immunoassay (EIA) antibody test to test plasma specimens from patients with confirmed *P. falciparum* infection.[14] Results in patients with confirmed malarial infection indicated that the combination of tests detected more positives than either test alone. Again, there is concern whether even this combination can detect the very low levels of parasitemia often seen in donors; especially since small amounts of blood are used.[14] PCR methods to detect plasmodium DNA or RNA may be the most sensitive (one parasite/50 µL) and specific but are technically demanding and the most expensive. One study, however, has indicated even this method may not be able to detect organisms below the level of 10 parasites/10 µL blood.

## Babesiosis

Babesiosis is endemic in the Northeastern and parts of the upper Midwest USA. Following malaria, it is the most common transfusion-transmitted parasitic disease.[15,16] It is an intraerythrocytic parasite that like the malaria parasite can be transmitted not only by RBC transfusion but also by the few RBC present in a unit of platelets.[15] The organisms frequently associated with human infection, *Babesia microti* and *B. divergens*, are transmitted by the bite of a tick of Ixodes sp. The WA l-type has been linked to transfusion-transmitted babesiosis.[17]

Most naturally occurring infections are asymptomatic or exhibit mild features. In asplenic, elderly, or immunocompromised patients a severe malaria-like illness with hemolytic anemia and renal failure can occur. *B. microti* organisms can survive at 4°C and at 25 °C. One study demonstrated that organisms in blood stored at 4°C were viable at day 17; those in blood held at 25°C were viable for 3 days.[18] Results from a study that monitored Babesia-infected subjects every 3 months for up to 27 months demonstrated organisms in the circulation (blood smear) for approximately a week but PCR assays for circulating babesial DNA were positive for 82 days.[19]

As with malaria and Chagas’ disease, there are no approved serological tests for donor screening and donor questions may not always elicit correct history since the infection is often asymptomatic.[19] Current diagnostic tests for babesiosis are not suited to large-scale donor screening. Examination of peripheral blood smears, indirect fluorescent antibody tests, PCR for detection of *B. microti* specific targets, or inoculation of animals are slow, costly, and/or labor-intensive methods. Efforts are underway to develop EIA tests for detection of babesial antibodies that might be suitable for donor screening.[20] At present any donor with a history of babesiosis is indefinitely deferred because of the possibility of persistent parasitemia.

## Chagas’ disease (American trypanosomiasis)

Chagas’ disease, caused by *Trypanosoma cruzi* is endemic in Central and South America and parts of Mexico. It is transmitted by a bug of the Reduviidae family. The disease is initially acquired when the infective trypomastigote stage is deposited on human skin in the insect’s feces after it takes a blood meal. The organism enters the human circulation through a break in the skin. The acute stage of the illness is short-lived and characterized by fever, anorexia, hepatosplenomegaly, and circulation of the trypomastigote form in blood. About 10% to 30% of those infected will develop chronic trypanosomiasis with intracellular invasion by the organism.[21] This intracellular amastigote stage is responsible for the chronic form of the disease which is characterized by neurological disorders, progressive damage to heart muscle, resulting in cardiomyopathy, or damage to the digestive system, resulting in megacolon or megaesophagus. During the chronic stage, infective trypomastigotes circulate in low numbers in the individual’s blood and make the blood potentially capable of transmitting the disease by transfusion.

In endemic areas the seroprevalence of the disease varies from less than 1% to 62% (depending on the country) with estimates of 16 to 18 million persons infected overall.[22–25] Blood donors in endemic areas are commonly tested for antibodies before donation and the risk of acquiring transfusion-transmitted Chagas’ disease from seropositive donors in endemic area ranges from 12% to 48%.[21,26] Transfusion transmission is the second most common method of acquiring the disease, followed by transplant-transmitted and finally transplacental (congenital). There have been four reported cases of transfusion-transmitted Chagas’ disease in the USA and two in Canada, with the majority due to *T. cruzi* contaminated platelets. The recipients were all immunocompromised and all but one of the donors was from a country endemic for *T. cruzi*.[27]

Despite the few documented cases of transfusion-transmitted *T. cruzi* infection, there is concern about the safety of the USA blood supply because of increased immigration from endemic areas. It is estimated that 25,000 to 100,000 Latin American immigrants in the USA may be infected with *T. cruzi*.[28] In addition, trypomastigotes have been shown to remain viable in stored whole blood for seven days, in platelets for 4 days, and in RBCs for 2 days with PCR testing for *T. cruzi* DNA remaining positive throughout the storage of the units.[29] Acute infection is usually diagnosed by observation of the trypomastigotes on a Wright-stained blood smear. However, in the chronic stage the circulating level of trypomastigotes is too low to be detected and therefore seropositivity is used as evidence of infection. Serologic tests using ELISA methodology are sensitive and specific in detecting parasitemia when seroprevalence of the organism is relatively high but they cannot readily distinguish between acute and chronic infection.[27] Another problem with current serologic tests is that the antigens used are derived from whole organisms and some antigens may be shared with other parasites such as *Leishmania* sp. This yields cross-reactions and false positive results, which may be of more concern in areas such as South and Central America where leishmaniasis is also endemic. A serologic test using four recombinant *T. cruzi* antigens was evaluated and showed greater than 99% sensitivity, greater than 98% specificity and that it could be used in blood donor screening.[30]

## Leishmania

*Leishmania donovani*, the etiologic agent of visceral leishmaniasis is transmitted by the bite of a sandfly. The organism is an intracellular parasite that is present primarily in cells of the reticuloendothelial tissue and cells of the mononuclear phagocytic system. Literature search showed only 11 reports of transfusion-transmitted leishmaniasis. Of these, 10 were individual case reports and in one paper from Brazil 32 cases of kala-azar were reported out of 82 patients undergoing hemodialysis.[31] All 10 individual case reports were from Asia and Europe. Out of the 10 reports, five were infants and four patients were children of less than 6 years age. Only one adult case of transfusion-transmitted leishmaniasis was reported in a 30 year old female from Haryana, India.[32] The time between the transfusion of the *Leishmania* infected blood and first clinical manifestation was available in 10 reports; and the mean incubation period was 7.4 + 5 months.

Screening of donated blood by microscopic examination is not a sensitive tool and aspirates from the spleen or the bone marrow will be unethical. Immunodiagnostic testings, including ELISA using recombinant antigens such as rK-39 developed from *Leishmania chagasi* of the new world or a recently developed recombinant antigen rKE16 from *Leishmania donovani* from India[33,34] and PCR technology can be used for mass screening of donor blood samples. But these methodologies may have financial and technical difficulties. It may be suggested that, all donors be screened for specific antileishmania antibodies. A rapid test using rKE16 antigen developed by us with the financial support from department of biotechnology is now commercially available at a very economic price.

## Toxoplasma

Toxoplasmosis is a zoonosis caused by *Toxoplasma gondii*, a parasite that is hosted in cats and dogs and has three forms: trophozoites, cysts, and oocysts. *T. gondii* is transmitted through several routes: ingestion of *T. gondii* oocysts, eating undercooked contaminated pork or beef, direct contamination of open wounds, and vertical transmission from mother to infant. In addition, the agent has been reported to be transmitted through blood transfusion and organ transplantation.[35] Nevertheless, the risk of *T. gondii* transmission through blood transfusion is extremely low, and serologic testing of antibodies to *T. gondii* in blood donors appears to be unnecessary. It has been suggested that people who are at increased risk of toxoplasmosis, such as immunosuppressed individuals and pregnant women, receive *T. gondii* antibody-negative blood components for transfusion. The universal program of leukodepletion that is currently carried out in Canada may reduce the risk of transfusion-transmitted toxoplasmosis.

## Microfilariae

Microfilariae can be transmitted by blood transfusion and they may be circulated in the recipient’s blood but they do not develop into adult worms. Mortality associated with transfusion-associated filarial infection is not documented but it may give rise to morbidity in transfusion recipients in terms of allergic reaction. In a study[36] carried out to investigate the association of post-transfusion reactions and filarial infections in an endemic area out of about 11, 752 transfusion recipients that were followed up in 15 months period, 47 (0.4%) post-transfusion reactions (PTR) were reported. Blood donors with active history of filarial infection should be deferred from donating blood. Filarial antigen detection test may be employed as screening test for blood donors, if possible.

## Inactivation of parasites

The concept of pathogen inactivation in blood components is to reduce the residual risk of known pathogens and to effectively eliminate new, yet unknown pathogens. However, the different approaches should increase the blood safety without compromising the product efficacy or causing adverse effects, as toxic or mutagenic chemicals may be used in the process. The choice of a pathogen reduction approach depends on whether it is used to treat components for transfusion such as RBC, PLT and plasma, or for products manufactured from the plasma. In Europe, two distinct methods, methylene blue (MB) and solvent-detergent (SD) are currently employed for the treatment of plasma intended for transfusion. MB is a phenothiazine colorant that inactivates most viruses and bacteria after exposure to visible light. While it has the advantage of being useful for single plasma units, its ineffectiveness against intracellular pathogens and probable interaction with coagulation factors considerably reduce its efficacy.[37] The SD approach acts by disrupting the envelope proteins of targeted pathogens, thus compromising the integrity of the pathogen and rendering it non-infectious. This approach is used on small pools of plasma. The limitation of this technique is that it is not active against non-enveloped pathogens, and that levels of coagulation factors such as protein S may be decreased significantly by some of the SD treatment methods.[38] Amotosalen HCL (S-59) is a synthetic psoralen which, when combined with exposure to ultraviolet A [UVA] light, causes a permanent crosslink in bacterial and viral nucleic acid chains, thereby stopping pathogen replication.[39] Extensive studies have shown that this approach is effective against all pathogens that are currently screened for, including protozoans (*T. cruzi*).[40]

Donor leukocytes, the residual white blood cells in platelet or red cell transfusions, are associated with potential adverse reactions. Leukocytes, with their specific allogeneic structure (exposing the HLA class I and class II antigens on their surface) are main targets of the recipient’s immune system. Some transfusion recipients develop a fever after transfusion in response to the donor leukocytes. Repeated exposure to donor leukocytes may create an immune response that makes the recipient refractory to the donor platelets, thereby deriving no benefit from the transfusion event. In addition, some infectious agents are transmitted in donor leukocytes, in which they reside. In an effort to overcome these adverse effects, methods of removal of the donor leukocytes---leukoreduction or leukodepletion---have been developed. Today’s technology permits removal of > 99.99% of donor leukocytes, usually by means of filtration of the red cells and or platelets. The ability of leukocyte filters to remove contaminating bacteria or protozoa, such as *Trypanosoma cruzi*, the agent causing Chagas disease, from donor blood is questionable.[41]

Although the risk of transfusion-transmitted infections today is lower than ever, the supply of safe blood products remains subject to contamination with known and yet to be identified human pathogens. Only continuous improvement and implementation of donor selection, sensitive screening tests and effective inactivation procedures can ensure the elimination, or at least reduction, of the risk of acquiring transfusion transmitted infections. In addition, ongoing education and up-to-date information regarding infectious agents that are potentially transmitted via blood components is necessary to promote the reporting of adverse events, an important component of transfusion transmitted disease surveillance. Thus, the collaboration of all parties involved in transfusion medicine, including national haemovigilance systems, is crucial for protecting a secure blood product supply from known and emerging blood-borne pathogens.
